# Hardy Weinberg Equilibrium Disturbances in Case-Control Studies Lead to Non-Conclusive Results

**DOI:** 10.22074/cellj.2021.7195

**Published:** 2020-04-22

**Authors:** Jose Luis Royo

**Affiliations:** Department of Surgery, Biochemisty and Immunology, School of Medicine, University of Malaga, Boulevar Louis Pasteur s/n, Málaga, Spain

## Abstract

Recently, it has been proposed the association of a common deletion affecting toll-like receptor 2 promoter (-196 to -177) to type 2 diabetes mellitus risk. However, genotyping results show a significant deviation from the HardyWeinberg Equilibrium (HWE). The law of Hardy-Weinberg shows that for an autosomal biallelic marker with allele frequencies f_A_=p and f_a_=q, the proportion of subjects with genotypes AA, Aa, and aa should follow the following: f_AA_=p^2^ , f_Aa_=2pq, and f_aa_=q^2^ . Departure from HWE or Hardy-Weinberg Disequilibrium (HWD) in a human control population can be caused by natural factors such as selective pressure against a certain genotype. However their prevalence is scarce and magnitude of effect over the HWE are small. Other factors such as inbreeding caused by consanguinity, population stratification, or technical problems in genotyping are more usual. Nevertheless, if the control population follows a perfect HWE, the presence HWD among patients might be explained by the genetic association and evidencing a real link between the locus and the trait under study. However, HWD affecting both cases and controls, such as the one reported might be explained by one of the aforementioned issues.

Genetic case-control studies have been proven as a powerful strategy to decipher the biochemical pathways underlying complex diseases. The basis of this approach is to find whether patients share an ancestral haplotype harboring either a common risk factor or directly a causative mutation. This implies sharing a determined genotype due to the fact of being patients (identical by state) rather than being relatives (identical by descent). Geneticists determine the genotypes and then compare allele frequencies in unrelated patients and control series. Whenever the mutant allele is in linkage disequilibrium with the causative mutation, we observe a statistically significant increase on its allele frequency among cases. However, independent of the allele frequency and the locus under analysis, any genetic marker mapping the autosomal chromosome shall follow the Hardy-Weinberg Equilibrium (HWE) (1, 2). This apparently simple rule states that for a biallelic locus with frequencies p and q respectively, genotypic frequencies must follow the p^2^, 2pq and q^2^. Disturbances of the Hardy-Weinberg Equilibrium (HWD) occur when natural selection operates over a particular genotype giving a differential fitness to any of them, such in the case of the hemoglobin (Hb) locus, associated to sickle cell anemia and resistance to *Plasmodium falciparum* infection (3). However, these cases arescarce in the literature, and large series are required to find statistically significant results. More often, HWD evidences population stratification. If a study series comprises subjects with different genetic background differing in their allele frequencies, their mix would exhibit a HWD. This is the called Wahlund effect and happens when each population, independently, fit HWE (4). Alternatively, HWD might be due to the existence of inbreeding in the series. This is evidenced by a reduction of the heterozygosity within a population (5). Finally, the HWD might be consequence of genotyping problems. A cross contamination typically pops up due to an excess of heterozygotes, while a reduced sensitivity of the mutant allele results in a lower mutant homozygotes frequency. Alternatively, the inclusion of duplicates in the genotyping series might also be associated to HWD.

Recently, Ermi؛ Karaali et al. (6) studied the role of a common Toll-like receptor 2 promoter deletion (196 to-177) in type 2 diabetes risk (T2DM). The rational underlying work roots on the contrasted role of TLR2 on human innate immunity. They performed a casecontrol study determining the presence of this promoter deletion among 100 cases and 98 age-matched controls, and concluded that the deletion allele was associated to T2DM risk. However, this conclusion should betaken with caution. Both cases and controls show a remarkable HWD (X^2^ P=4.99×10^-8^ and 1.4×10^-4^ for cases and controls, respectively). The nature of this HWD relies on an underrepresentation of heterozygotes in both cases and controls that can be illustrated in the De Finetti diagram (Fig.1). The observed heterozygotes among controls only represent 61% from what expected according to HWE (3 vs. 4.87) and this effect was more accused within the T2DM series with only 45.5% from what expected (14 vs. 30.78). Therefore inbreeding coefficient estimates range between F=0.545 and F=0.384 (cases and controls, respectively). *TLR2* -196 to -177 has been extensively determined in different studies with no evident HWD and, whenever reported, this was constrained to cases (Table 1). It has been recently reported that besides using the information from 2,405 subjects available from the 1,000-genome project release 3, no HWD is found for control populations of Europe, Africa, Asia or America at this locus (7). This suggests that Ermi؛ Karaali et al. (6) might have fallen in at least one of the aforementioned problems associated to HWD. It should be highlighted that we cannot rule out the possibility that functional variants affecting TLR2 physiology might be associated to T2DM. However, this potential role of *TLR2* -196 to -177 deletion shall be repeated following the adequate controls in order to determine its potential implication in T2DM risk.

**Fig.1:**
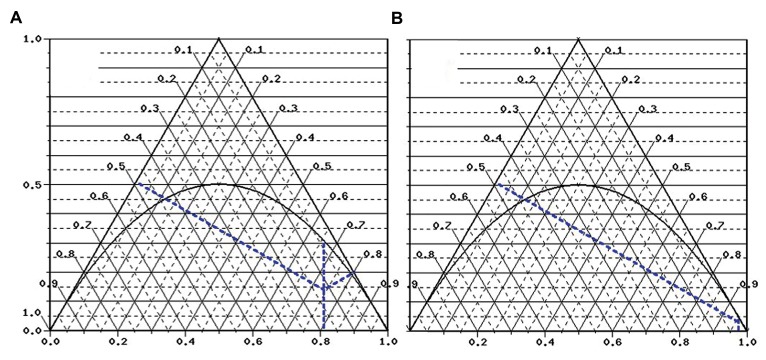
De Finetti representation of Ermi؛ Karaali et al. (6) results of TLR2 -196 to -177 variant on T2DM risk. De Finetti representation (8) of **A**. Both cases and **B**. Controls from Ermi؛ Karaali et al. (6). The X-axis represents the frequency of the Ins allele. The intersection of the parabola and vertical line represents the frequency of genotype Ins/Del under f Hardy-Weinberg Equilibrium.

**Table 1:** HWE was assayed using the freely available resource at https://ihg.gsf.de/cgi-bin/hw/hwa2.pl from the University of Munich. P values were calculated using Pearson’s goodness-of-fit chi-square with one degree of freedom


Disease	Subjects	Ins/Ins	Ins/Del	Del/Del	MAF	HWE	Country	Reference
						P value		

T2DM	Controls	94	3	1	0.03	1.42×10^-4^	Turkey	(6)
	Cases	74	14	12	0.19	4.99×10^-8^		
Parkinson’s disease	Controls	95	21	2	0.11	0.511	Greece	(9)
	Cases	156	52	7	0.15	0.309		
VIH susceptibility	Controls	189	65	3	0.14	0.318	Spain	(7)
	Cases	160	18	0	0.05	0.477		
General population	Controls	208	85	11	0.18	0.531	Poland	(10)
Gastric cancer	Controls	75	65	8	0.27	0.202	Japan	(11)
	Cases	126	112	51	0.37	4.1×10^-3^		
Alzheimer’s disease	Controls	172	168	60	0.36	0.077	China	(12)
	Cases	150	161	89	0.42	4.37×10^-4^		

